# Data in support of energy performance of double-glazed windows

**DOI:** 10.1016/j.dib.2016.03.094

**Published:** 2016-04-04

**Authors:** Mahmoud Shakouri, Saeed Banihashemi

**Affiliations:** a School of Civil and Construction Engineering, Oregon State University, 220 Owen Hall, Corvallis, OR 97331, United States; b School of Built Environment, University of Technology Sydney, 15 Broadway, Ultimo, NSW 2007, Australia

**Keywords:** Window, Energy efficiency, Residential buildings, Energy, Optimization, Energy savings

## Abstract

This paper provides the data used in a research project to propose a new simplified windows rating system based on saved annual energy (“Developing an empirical predictive energy-rating model for windows by using Artificial Neural Network” (Shakouri Hassanabadi and Banihashemi Namini, 2012) [1], “Climatic, parametric and non-parametric analysis of energy performance of double-glazed windows in different climates” (Banihashemi et al., 2015) [2]). A full factorial simulation study was conducted to evaluate the performance of 26 different types of windows in a four-story residential building. In order to generalize the results, the selected windows were tested in four climates of cold, tropical, temperate, and hot and arid; and four different main orientations of North, West, South and East. The accompanied datasets include the annual saved cooling and heating energy in different climates and orientations by using the selected windows. Moreover, a complete dataset is provided that includes the specifications of 26 windows, climate data, month, and orientation of the window. This dataset can be used to make predictive models for energy efficiency assessment of double glazed windows.

**Specifications Table**

**Table:** 

Subject area	*Civil Engineering*
More specific subject area	*Energy*
Type of data	*Tables, Figures*
How data was acquired	*Technical datasheets, simulation in EnergyPlus*® [3]
Data format	*Raw and modified*
Experimental factors	*Window type, climate data and building orientation.*
Experimental features	*A* 26×4×4 *full factorial design was conducted resulting in* 416 *simulations.*
Data source location	*Ardabil* (*cold climate*), *Bandar Abbas* (*tropical climate*), *Rasht* (*temperate climate*), *and Yazd* (*hot-arid climate*); *all from Iran.*
Data accessibility	*Data are within this article.*

**Value of the data**

• Presenting a comprehensive dataset monthly and annual energy performance of double-glazed windows faced to different orientations and located in diverse climates.
• Understanding and exploiting the methodological approach to assess energy consumption of various building components.
• Facilitating future studies on windows energy performance optimization by the data included here.

## 1 Data

Simulated annual energy for each window is provided in the Supplementary materials (AnnualEnergy.xlsx). The file consists of 4 tabs in which each tab represents one climate.

The dataset used for developing a neural network model [1] is given in the Supplementary materials (ANN.xlsx). This dataset contains 1200 rows of data which represents all the possible combination of variables used to develop a window rating system [1], [2]. The file includes information related to U-factor, solar heat gain coefficient (SHGC), visual transmittance (VT), emissivity of each window, average monthly temperature, average monthly percent humidity, average monthly wind speed, average monthly direct and diffuse solar radiation, orientation, month and monthly saved energy.

## 2 Experimental design, materials and methods

**Fig. 1:**
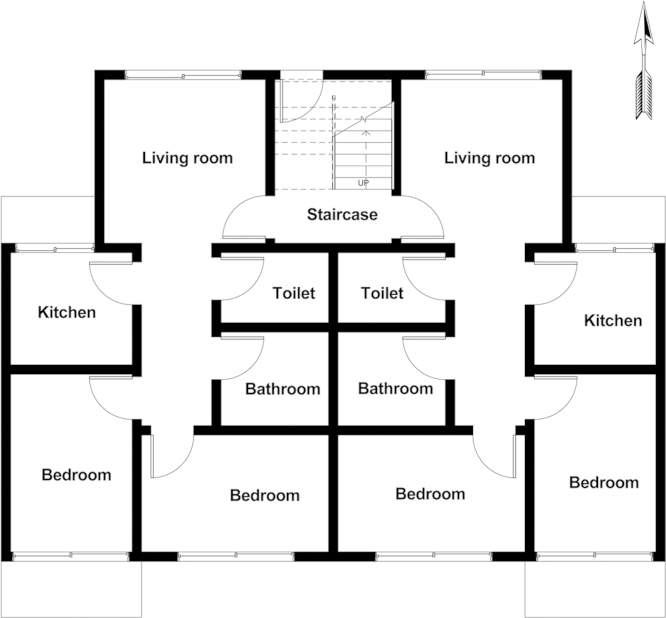
Sample layout of the four-story residential building used in the simulation.

**Fig. 2:**
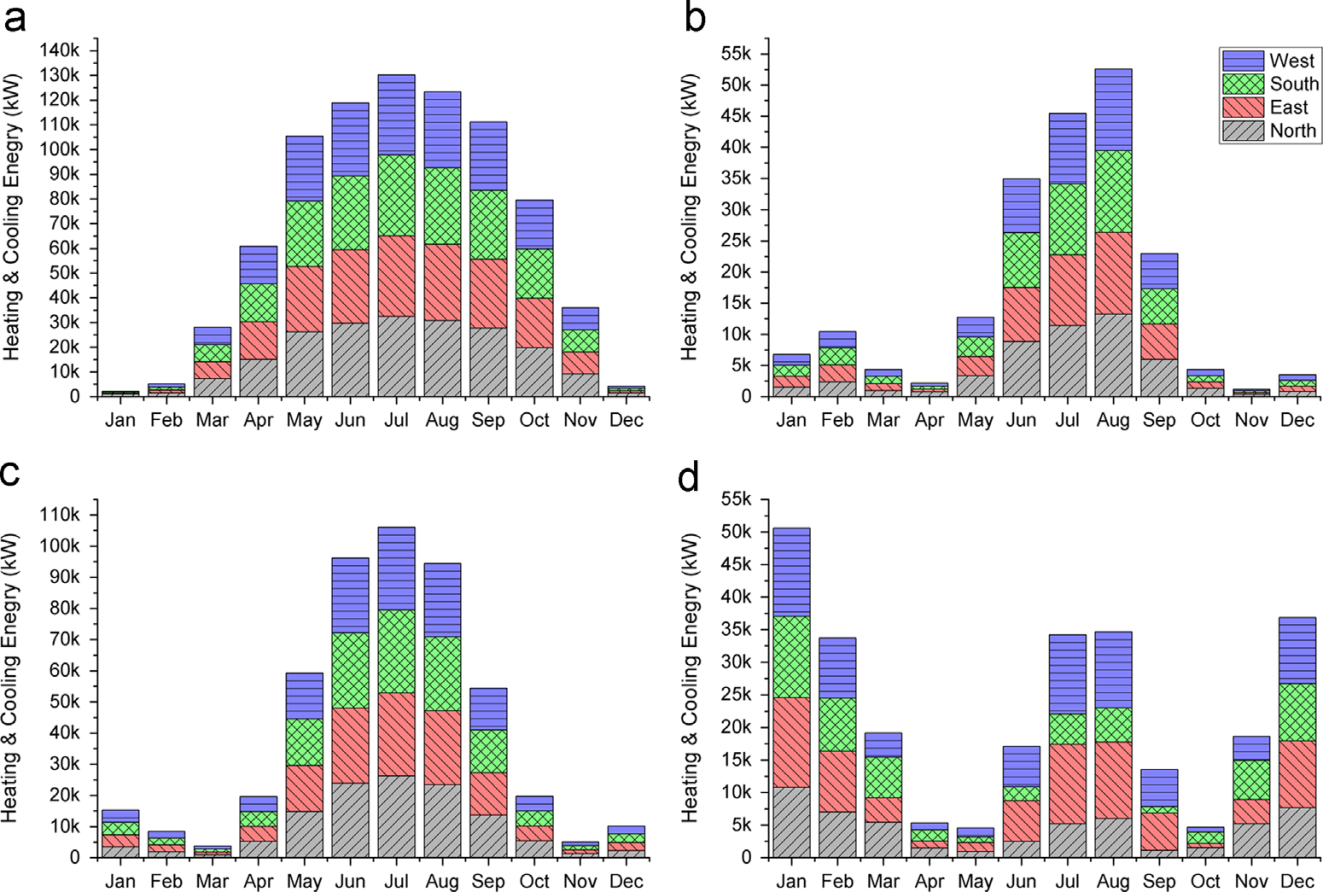
Monthly heating and cooling energy required for different orientations in four climates: (a) tropical; (b) temperate; (c) hot and arid; and (d) cold.

A 26×4×4 full factorial design with window type, climate, and building orientation as independent variables was used in this data article. A four-story residential building with the gross area of 527 m^2^ was designed in Revit (Fig. 1) and exported to Energyplus for energy simulation [3]. The ratio of total windows area to the floor area was 22.8%. To measure the effects of windows on energy consumption, the properties of other materials such as roof, floor, etc. were held constant and the annual energy required for keeping the building within the comfort band of 18–24 °C was simulated by varying the window types. Four climates of cold, tropical, temperate, and hot and arid were selected to represent common climate types. In each climate, the main facade of the building was rotated to face the main four orientations of North, West, South and East. 416 simulations were run (26 windows×4 climates×4 orientations) to calculate annual energy consumption to keep the building within the comfort band. In order to develop a simplified rating system a neural network model (ANN) was developed by considering the physical properties of windows (i.e., U-factor, solar heat gain coefficient (SHGC), visual transmittance (VT), and emissivity of each window), climate data (i.e., dry bulb temperature, percent humidity, wind speed, and direct and diffuse solar radiation), and the orientation of building as inputs. For the output, the required monthly energy of the control window (a single glazed window) was subtracted from the required monthly energy of each type of double glazed window, resulting in the monthly saved energy. Fig. 2 shows the monthly required energy by using the control window in different climates and orientations.

## Supplementary data

Please find supplementary tables in the online version.

## Footnotes

Appendix A Supplementary data associated with this article can be found in the online version at doi:10.1016/j.dib.2016.03.094.
